# Therapeutic Effect of Movement Control Exercises Combined With Traditional Physiotherapeutic Rehabilitation in A Patient Suffering With Non-Specific Low Back Pain: A Case Report

**DOI:** 10.7759/cureus.61868

**Published:** 2024-06-07

**Authors:** Nikita Kaple, Pratik Phansopkar, Manali A Boob

**Affiliations:** 1 Musculoskeletal Physiotherapy, Ravi Nair Physiotherapy College, Datta Meghe Institute of Higher Education and Research, Wardha, IND

**Keywords:** flexibility, muscle strength, nonspecific low back pain, quality of life (qol), movement control exercise

## Abstract

Low back pain (LBP) is a common complaint among individuals engaged in physically demanding occupations, such as construction workers, luggage lifters, manual laborers, and drivers. One of the main problems facing modern healthcare is treating these people. The identification of distinct patient subgroups with non-specific LBP and the development of specialized, more effective therapies are of crucial significance to enhancing evaluation and treatment regimens. This case report describes the evaluation and management of non-specific LBP in a male construction worker who complained of severe low back discomfort. Enhancing the muscular endurance, strength, and flexibility of the back muscles and soft tissues is the main goal of exercise therapy, which is the key to the management of nonspecific LBP. This patient receives a four-week treatment regimen that includes movement control exercises and several advanced therapeutic modalities. The direction of movement control ensures the way patients sit when their back muscles contract. Back muscle activation rates are greater in the active extension group and lower in the flexion group. A comprehensive rehabilitation program that was effective for our patient, who was experiencing lower back discomfort. We assessed the efficacy of our outcome measures using a variety of outcomes, including the modified Oswestry disability index, visual analog scale, range of motion, Quebec back pain disability scale, and pressure biofeedback unit for muscle strength. In addition to a standard physiotherapy course, providing modern physiotherapeutic treatments was found to be more beneficial for enhancing the patient's overall health and quality of life.

## Introduction

Low back pain (LBP) is of two types: specific and non-specific. Specific LBP is the term used for pain that results from a particular disease, structural problem with the spine, or pain that transmits to another part of the body. Non-specific LBP occurs when specific ailments or anatomical motives cannot be identified as the source of the pain [1]. The most common musculoskeletal ailment in the world and the primary cause of disability is LBP. This is the condition that has the highest potential for rehabilitative benefits [1]. The pain lacks a clear origin and cannot be linked to a particular incident or illness [2]. With a global point prevalence of 7.3% and a one-year point prevalence of 38%, LBP affects 540 million individuals globally at any given moment [3]. Physical risk factors (such as heavy lifting and extended standing or walking) increase the chance of experiencing an episode of nonspecific LBP. A bad lifestyle (such as obesity), psychological issues (such as depression and work unhappiness), and past instances of LBP are additional risk factors [4]. Older adults and those with other chronic illnesses are more likely to suffer from osteoporotic fractures, especially if they have used oral steroids for an extended period of time [5].

Individuals with motor cognitive impairment (MCI) incite discomfort by engaging in maladaptive physical and cognitive compensatory behaviors for their illnesses, leading to persistent pain. The underlying cause of the pain condition in these people is an impairment in movement control. These individuals may be knowingly aggravating their discomfort because they are unable to control their movements appropriately [6]. Individuals with movement impairment (MI) and movement control impairment are further divided into patients with mechanical, non-specific LBP. Individuals with MI may have limitations to their range of motion in one or more directions. A deficiency in motor control during functional routines is known as a motor control deficit [7]. Exercises are chosen according to the impairment's direction, such as in the frontal plane, flexion, and extension. Patients must first figure out ways to manage their lumbar spine's position and mobility in an array of postures, including standing, squatting, four-point kneeling, and sitting. The management of this condition has proven the efficacy of several conservative treatments, which are recommended before considering surgical interventions. These usually include soft tissue treatment, lumbar stabilization exercises, McKenzie or end-range loading exercises, pelvic blocks, spinal manipulations or mobilizations, and movement control exercises [8]. Physiotherapy is the major intervention for conservative treatment, and it uses a number of modalities and therapeutic strategies to restore function while also strengthening and stabilizing the spine.

The goal of this case report was to investigate the effectiveness of a physical therapy rehabilitation program for a 35-year-old male referred for non-specific LBP. Non-specific back pain lacks a definite clinical origin and frequently causes profound discomfort, restricted movement, and functional restrictions. This health condition is especially difficult for those in physically demanding jobs since it hinders their capacity to do critical duties. The technique, which combines movement control exercises with standard physiotherapy, seeks to address these difficulties thoroughly. Movement control exercises are intended to improve the coordination and strength of the muscles that support the spine, resulting in increased stability and less discomfort. To enhance the quality of life of the patients, the therapeutic alternatives that were used in this report included posture control exercises, aerobic training, upper and lower extremity strengthening activities, and movement control exercises [9].

## Case presentation

A 37-year-old male who works as a construction worker, with a height of 152 centimeters, a weight of 60 kilograms, and a body mass index of 26 kilograms per square meter, presented with a history of low back discomfort lasting two months to the Orthopedic Department of Acharya Vinoba Bhave Rural Hospital, India. The pain was dull and aching, primarily localized to the lumbar region, exacerbated by prolonged standing, lifting heavy objects, and bending forward. There was no relevant history of trauma present. He reported difficulty in performing his job duties due to the pain. A physical examination revealed tenderness in the lumbar paraspinal muscles and mild restriction in the lumbar range of motion. Imaging studies, including X-ray of the lumbar spine, were done but did not show any significant structural abnormalities or specific pathology. Non-specific LBP was diagnosed based on the clinical presentation and testing results. They had been prescribed medication. Physiotherapy sessions were advised by the orthopedic doctor. After a one-week course of prescribed medication, he experienced minimal relief from his symptoms. The physiotherapy sessions were continued.

Clinical findings

**Figure 1:**
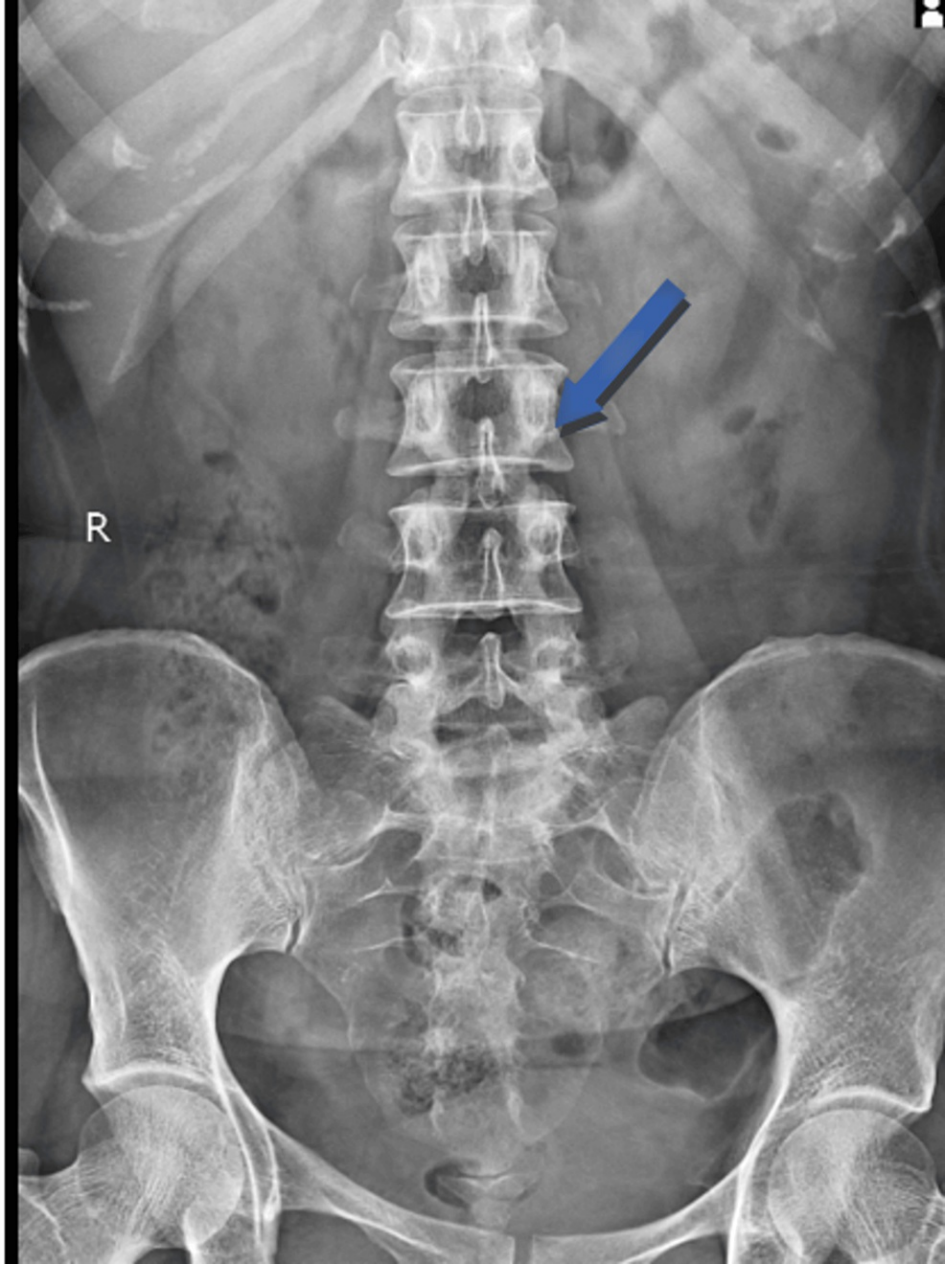
Demonstrates that the vertebral bodies of the lumbosacral spine have a normal density on a radiograph. Both the spinous and pedical processes are intact. The joint of the sacrum seems to be normal. No pathology has been shown.

Prior to the examination, the patient's permission was obtained. The patient was assessed while lying prone. The patient had lower-back paraspinal spasms on both sides. A thorough evaluation of the musculoskeletal system was conducted. On examination, there was grade 2 tenderness in the lumbar paraspinal muscles, particularly at the L4-L5 level. Range of motion tests indicated a slight limitation in lumbar flexion and extension at the level of L5. The straight leg raise test was negative, with signs of muscle weakness in the core and hip musculature. There were no signs of radiculopathy or significant neurological deficits. Range of motion was assessed using the modified Schober's test to measure lumbar flexion. The test revealed a restricted lumbar flexion of 1.8 centimeters, suggesting limited flexibility and potential impairment in lumbar spine mobility. Imaging studies, including X-rays of the lumbar spine, were done but did not show any significant structural abnormalities or specific pathology, as shown in Figure 1. The strength of the lumbar flexor and extensor muscles decreased. Muscle strength and lumbar stability were evaluated using a pressure biofeedback unit (PBU). The PBU assessed the activation and endurance of the core muscles by measuring their ability to maintain a neutral spine position during various exercises. The evaluation of the core strength shows a 90-millimeter mercury deflection. The patient demonstrated decreased muscle strength and stability, indicative of muscular weakness and poor core control. The modified Oswestry disability questionnaire (MODQ) was administered to determine the impact of low back pain on daily activities and functional ability. The patient scored 46% on the MODQ, indicating moderate disability and limitations in performing routine tasks due to low back pain. Table 1 presents the timeline of events.

**Table 1:** Timeline of events

Sequence of events	Dates of events
A patient came to the orthopaedic department with a complaint of low back discomfort.	18 /01/2024
A radiological analysis was conducted. Nonetheless, a diagnosis of non-specific low back pain was made because neither the clinical presentation nor the diagnostic tests revealed anything.	18 /01/2024
A patient came to the physiotherapy department.	25/01/2024
A physiotherapy rehabilitation session was started.	25/01/2024
End of physiotherapy intervention and advice home program.	22/02/2024

Therapeutic interventions

**Figure 2:**
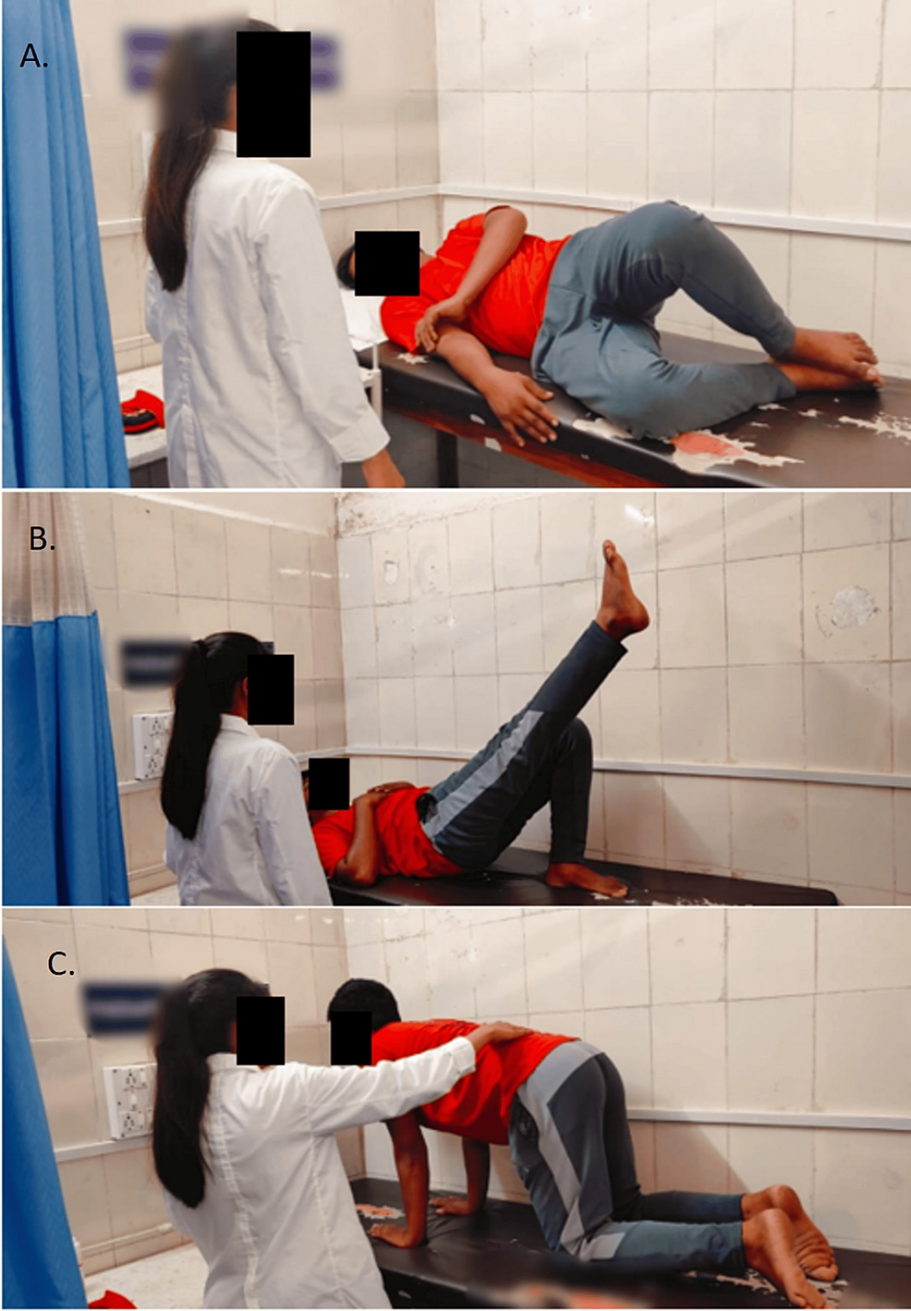
Patients performing movement control exercises A: clamshell, B: unilateral bridging, C: rocking forward and backward

**Figure 3:**
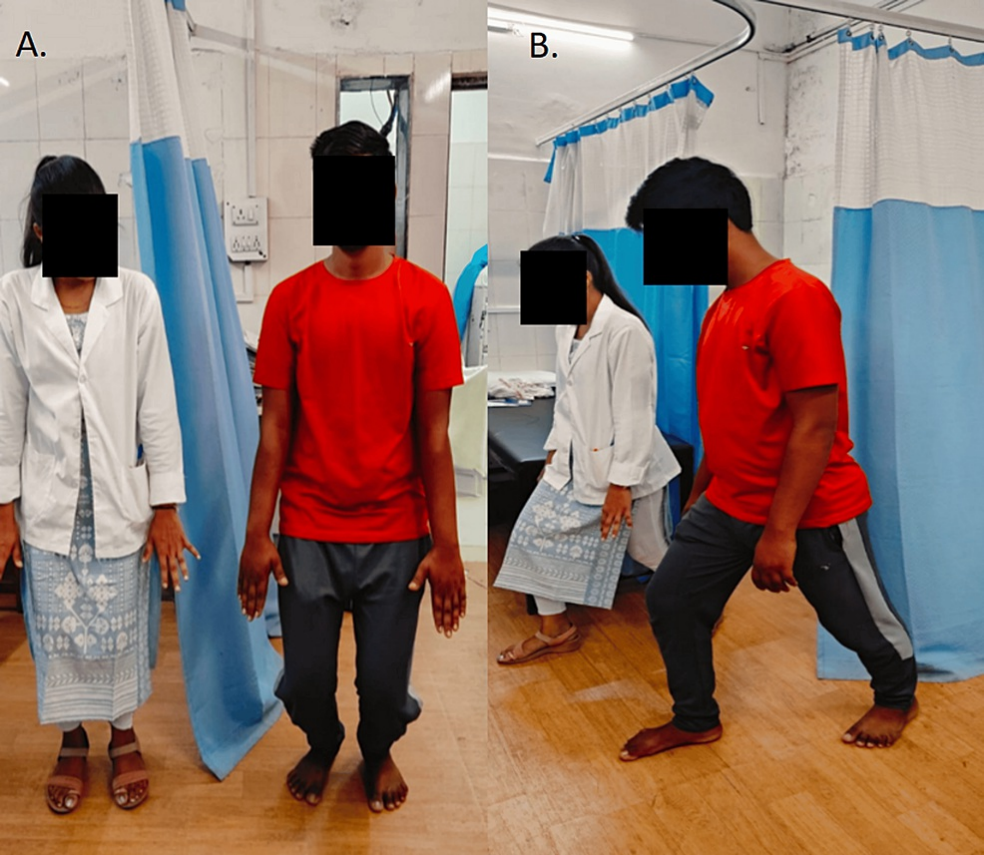
Performing another set of movement control exercises A: squatting, B: forward lunges

Physical therapy sessions were initiated to help correct muscle imbalances, enhance posture, and encourage healthy biomechanics. To prevent symptoms from worsening during work-related tasks, correct body mechanics, ergonomic concepts, and activity moderation were taught. The core component of the treatment plan was a structured exercise program aimed at improving lumbar spine stability, flexibility, and strength. This program included exercises targeting the core muscles, lumbar extensors, and hip flexors to correct muscle imbalances and promote functional ability, manual therapy procedures, and techniques, such as joint and soft tissue manipulation, were added to reduce lumbar spine stiffness and enhance mobility. Table 2 illustrates low back pain rehabilitation. Figures 2 and 3 show the movement control exercises.

**Table 2:** Therapeutic intervention for an individual suffering from nonspecific low back pain

Goals	Therapeutic intervention	Rationale
Patient education to prevent low back pain	Exercises and postural changes throughout the day can minimize and partially reverse the stresses imposed on the back. Educating the individual on the way to manage their symptoms with certain postures and trunk motions, modifying functional activities to alter trunk movement and alignment patterns, and many other techniques.	Teaching proper lifting techniques and ergonomics reduces strain on the back and prevents future injuries.
To improve the patient's well-being and alleviate their lower back discomfort	Treatment modalities like heat therapy: The muscles become more relaxed the longer the heat is managed. The patient received lumbar interferential treatment while prone to alleviate discomfort and muscular spasms. For 10 minutes, four electrodes placed five by nine centimeters in a vector pattern were beat at a frequency of 50 Hz.	Heat promotes tissue repair and eases tense muscles by increasing blood flow to the damaged area.
Restoration of joint range of motion and soft tissue extensibility	Implement stretching exercises targeting tight muscles and connective tissues. Back discomfort stretching exercises, such as basic hamstring stretch. Exercises that include extension can alleviate neuronal stress. Flexion exercises stretch the dorsolumbar fascia and lessen the articular weight-bearing load on the facet joints.	Stretching helps improve flexibility, reduce muscle stiffness, and alleviate tension in the lower back.
To enhance postural alignment guidance for modifying lifestyle and ergonomic guidance	Physiotherapists also focus on offering precise ergonomic equipment and counseling patients to use the right tools at work to prevent and treat lower back pain. Recommendation to sit for extended periods on ergonomic seats for work. The patient keeps their back straight during squats while bending through the knees to raise enormous weights. Steer cautious of obvious waist-bending.	To avoid and treat low back aches by changing one's posture and utilizing the proper posture at work.
To enhance the lumbar spine's flexibility and strength	Movement control exercise was given focusing on core stability and control. Include exercises such as pelvic tilts, abdominal bracing, and hip hinges. Perform 2-3 sets of 10-15 repetitions for each exercise. Progress exercises gradually by increasing repetitions and sets, to avoid any possible strain or damage, these exercises were performed lightly and within a pain-free range of motion. Exercises are chosen according to the impairment's direction, such as flexion, extension, or frontal plane. Patients must first figure out ways to manage their lumbar spine's position and mobility in an array of postures, including standing, squatting, four-point kneeling, and sitting.	To maintain flexibility and to improve the strength and endurance of the abdominal and lumbar trunk musculature.

Outcome measures

**Figure 4:**
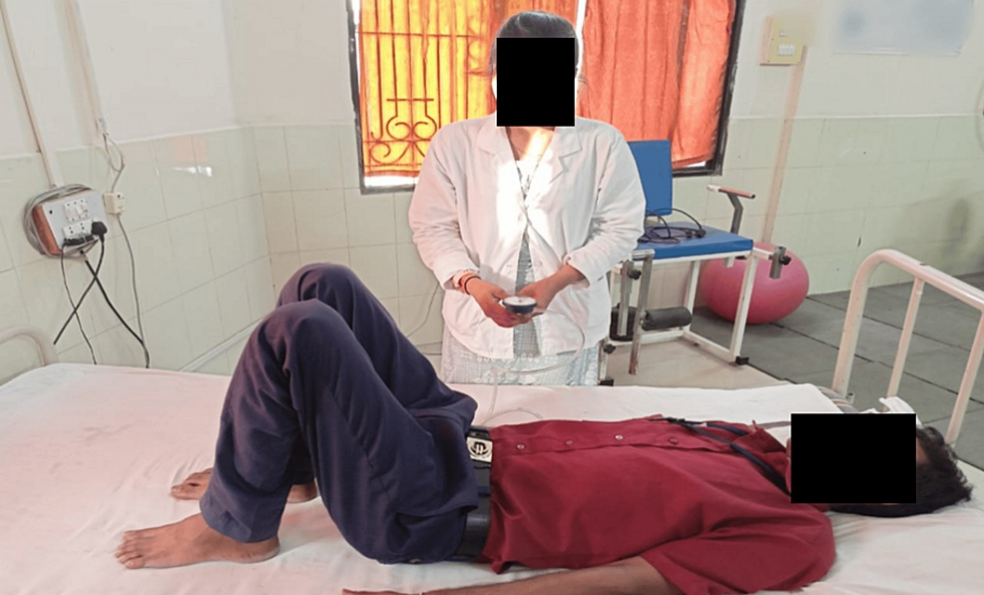
Measuring core abdominal strength via a pressure biofeedback

**Figure 5:**
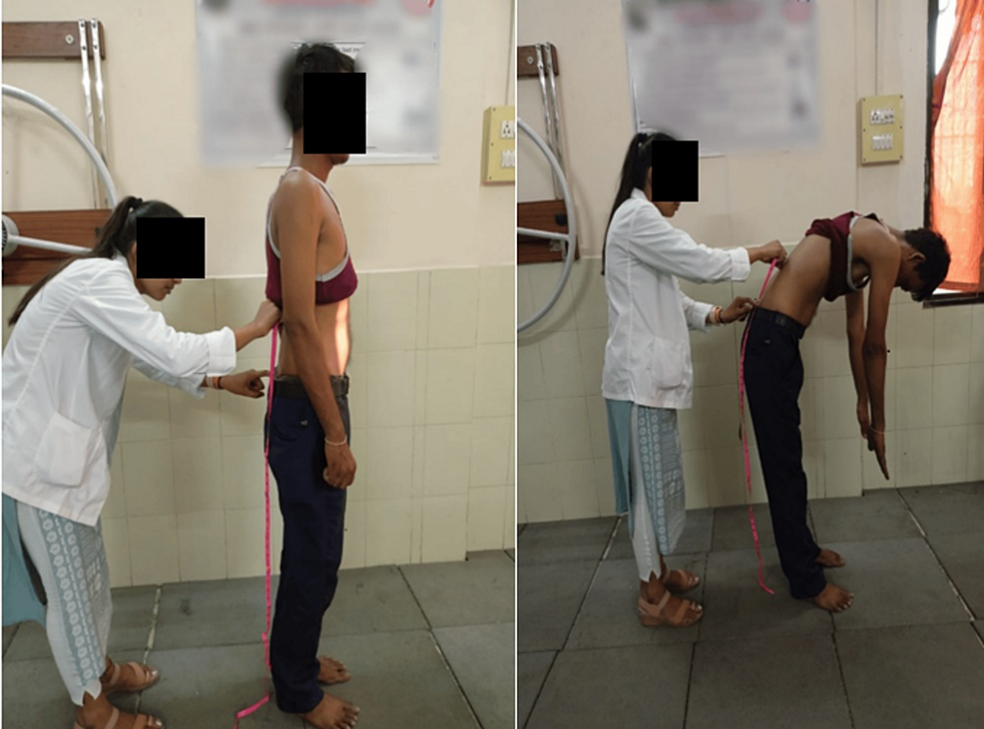
Measurement of lumbar flexion via an inch tape A: starting position, B: ending position

**Figure 6:**
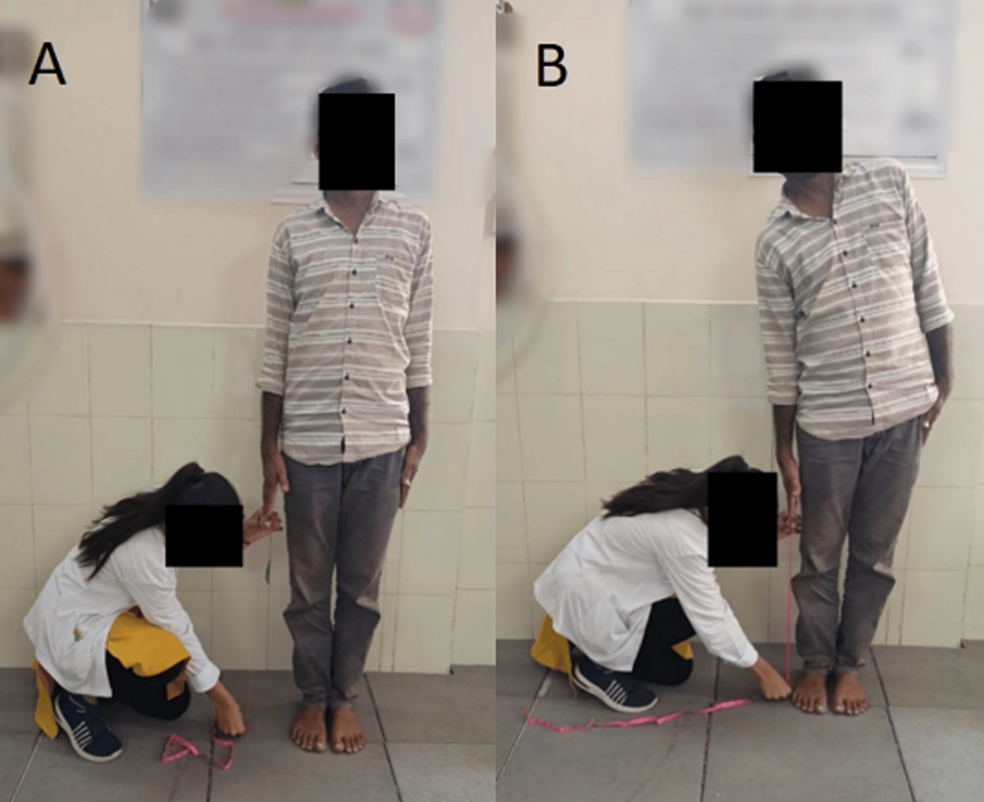
Measurement of lateral lumbar flexion via an inch tape A: starting position, B: ending position

Table 3 shows the pre- and post-physiotherapy rehabilitation outcome measures to evaluate the effectiveness of the given treatment protocol to the patients. Figure 4 shows the assessment of the core muscle strength with the use of pressure biofeedback. Figure 5 shows the lumbar flexion. Figure 6 shows the measurement of the lumbar lateral flexion via an inch tape.

**Table 3:** Outcome measures of pre- and post-physiotherapeutic interventions

Outcome measure	Pre-rehabilitation (before the start of physiotherapy treatment)	Post-rehabilitation (after four weeks of physiotherapy treatment)
Visual analog scale at rest	5.5 centimeters out of 10 centimeters	1.5 centimeters out of 10 centimeters
Visual analog scale at activity	6.8 centimeters out of 10 centimeters	2.4 centimeters out of 10 centimeters
Modified Schober's test to assess lumbar flexion	Shows a difference of 1.8 centimeters	Shows a difference of 4.5 centimeters
Range of motion of lateral lumbar flexion via an inch tape	17.5 cm from the tip of the middle finger to the floor	14.2 cm from the tip of the middle finger to the floor
Manual muscle testing for lumbar flexor	Grade 3+ (hold test position against slight pressure)	Grade 4 (hold test positioning against moderate pressure)
Manual muscle testing for lumbar extensor	Grade 3 (hold test position no added pressure)	Grade 4 (hold test position against moderate pressure)
To assess the strength of the core muscles via a pressure biofeedback unit.	45 mmhg	135 mmhg
Modified Oswestry disability questionnaire	21 out of 50 = 42% (severe disability)	3 out 50 = 6% of (minimal disability)
Quebec back pain disability scale	75 (greater disability)	15 (less disability)

## Discussion

Exercise treatment is administered to patients in the movement control group to improve lumbar spine movement control. One advised rationale for nonspecific LBP stands for movement control impairment. The latter is characterized by a change in the direction and alignment of the spine during movement [10]. Unlike motor control exercises, which embrace isolated deep trunk muscles that are strengthened and integrated into more complex static, dynamic, and functional exercises, the movement control method takes a different approach. Movement control exercises are described as controlling movement during everyday tasks. The cause of motor control exercise focuses mainly on pain and disability in patients with low back pain who have impaired spinal stability and control. The multifid and transversus abdominis are two examples of specific muscles whose function and performance are highlighted in motor control workouts [11].

The inadequate response to medication highlights the complexity of managing nonspecific LBP and underscores the importance of a multidisciplinary approach. While medications may provide symptomatic relief for some individuals, they may not address the underlying biomechanical factors contributing to LBP, particularly in individuals with physically demanding occupations like construction work. Physiotherapy plays a crucial role in addressing these factors through targeted interventions aimed at improving muscular strength, flexibility, and biomechanics [12]. Numerous therapies for the treatment of LBP are illustrated in a significant body of research. These interventions typically address limits in activity or involvement and impairments in body structure and function in individuals with NSLBP. Early physiotherapy or physical rehabilitation decreases the chance of transitioning from acute to subacute or chronic NSLBP [13]. European guidelines for the care of chronic non-SLBP patients advocate exercise therapy as a first-line treatment [14]. This is because high-quality data illustrate that exercise therapy is more effective than other strategies for treating this illness [15]. According to reports, people with LBP have a modified forward mechanism and neuromuscular dysfunction, as evidenced by delayed and less efficient contraction of the lumbar multifidus and transversus abdominis muscles during repeated trunk motions and dynamic limb activity [16]. It has been discovered that people with non-specific LBP-related impairment, kinesiophobia (the dread of pain with movement), and a lower quality of life are affected emotionally and cognitively [17].

## Conclusions

Physiotherapy is important for controlling nonspecific LBP because it relieves pain, increases strength, and improves balance. Early use of physiotherapy therapies is critical for maintaining strength and effectively managing pain. This case underscores the importance of a comprehensive and individualized approach to managing nonspecific LBP among construction workers. By addressing occupational risk factors, providing targeted interventions, and promoting self-management strategies, healthcare professional recovery enabled him to resume his job duties with improved functional capacity and quality of life. The case report found that movement control exercises improved nonspecific LBP outcomes such as range of motion and flexibility, pain relief, muscular strength, and quality of life. Patient-specific functional complaints and disability improved significantly after customized, personalized exercise regimens combined with traditional physiotherapeutic interventions.
